# Bone morphogenetic protein 10, a rising star in the field of diabetes and cardiovascular disease

**DOI:** 10.1111/jcmm.18324

**Published:** 2024-05-17

**Authors:** Xueyin Wang, Helin Sun, Haomiao Yu, Bingyu Du, Qi Fan, Baoxue Jia, Zhongwen Zhang

**Affiliations:** ^1^ Shandong Provincial Key Laboratory for Rheumatic Disease and Translational Medicine The First Affiliated Hospital of Shandong First Medical University & Shandong Provincial Qianfoshan Hospital, Department of Endocrinology and Metabology, The Third Affiliated Hospital of Shandong First Medical University Jinan China; ^2^ Department of Endocrinology and Metabology The Third Affiliated Hospital of Shandong First Medical University Jinan China; ^3^ Department of Endocrinology and Metabolism Affiliated Hospital of Shandong Second Medical University Weifang China; ^4^ Teaching and Research Section of Internal Medicine, College of Medicine Shandong University of Traditional Chinese Medicine Jinan China

**Keywords:** BMP10, cardiovascular disease, diabetic cardiomyopathy, endothelial dysfunction, immune inflammatory response

## Abstract

Early research suggested that bone morphogenetic protein 10 (BMP10) is primarily involved in cardiac development and congenital heart disease processes. BMP10 is a newly identified cardiac‐specific protein. In recent years, reports have emphasized the effects of BMP10 on myocardial apoptosis, fibrosis and immune response, as well as its synergistic effects with BMP9 in vascular endothelium and role in endothelial dysfunction. We believe that concentrating on this aspect of the study will enhance our knowledge of the pathogenesis of diabetes and the cardiovascular field. However, there have been no reports of any reviews discussing the role of BMP10 in diabetes and cardiovascular disease. In addition, the exact pathogenesis of diabetic cardiomyopathy is not fully understood, including myocardial energy metabolism disorders, microvascular changes, abnormal apoptosis of cardiomyocytes, collagen structural changes and myocardial fibrosis, all of which cause cardiac function impairment directly or indirectly and interact with one another. This review summarizes the research results of BMP10 in cardiac development, endothelial function and cardiovascular disease in an effort to generate new ideas for future research into diabetic cardiomyopathy.

## 1 INTRODUCTION

Cardiovascular disease (CVD) is a group of circulatory diseases that pose a major global hazard to human health. Several studies^1^, ^2^ indicate that CVD risk factors such as hypertension, diabetes, smoking and dyslipidaemia can cause endothelial cell injury, which contributes to the development and occurrence of CVD. The fact that the incidence of CVD is rising rapidly with economic and social development, particularly in developing nations, is of particular concern. Particularly, the growth of the diabetic population is associated with an increase in the prevalence of CVD, with diabetics having a significantly higher risk of cardiovascular events and cardiovascular mortality than non‐diabetics.^3^ Diabetes significantly increases the risk of cardiovascular events and cardiovascular mortality.

In recent years, the newly discovered cardiac‐specific growth factor bone morphogenetic protein 10 (BMP10) has come to the forefront of cardiovascular function and pathology research. After birth, BMP10 is predominantly expressed in the right atrium, and it plays a role in heart development and protection, according to studies.^4^, ^5^, ^6^ The absence of BMP10 can result in dysplasia of the ventricular wall. In contrast, increased BMP10 influences ventricular trabeculation and ventricular wall compaction.^7^ Additionally, BMP10 is a potent endothelial activator and has the ability to inhibit endothelial cell apoptosis.^8^ As the specific mechanisms of BMP10 action in humans for the heart and blood vessels have yet to be elucidated, we believe that a brief review of the signalling pathways of bone morphogenetic proteins and a focus on BMP10 research in the diabetic and cardiovascular fields will help advance our understanding of the pathogenesis of diabetic cardiomyopathy and encourage more researchers to pursue BMP10 research.

## 2 BMP10 SIGNALLING EXPRESSES BIOLOGICAL ACTIVITY THROUGH THE TGF‐β/BMP PATHWAY

As members of the transforming growth factor β (TGF‐β) superfamily, BMPs are multifunctional and pleiotropic, not only possessing strong inductive activity in bone, but also exerting broad roles in numerous cell types and processes.^9^ They play an important function in embryonic development and early skeletal development, as well as in the regulation of adult tissue homeostasis.^10^ More than 15 known BMPs are structurally related and can be categorized into four subgroups on the basis of amino acid similarity: BMP2/4, BMP5/6/7/8, BMP9/BMP10 and BMP12/13/14.^11^ At least six BMPs are expressed in the heart, including BMP2, BMP4, BMP5, BMP6, BMP7 and BMP10, whose distributions in the heart are distinct but overlapping.^12^

**FIGURE 1:**
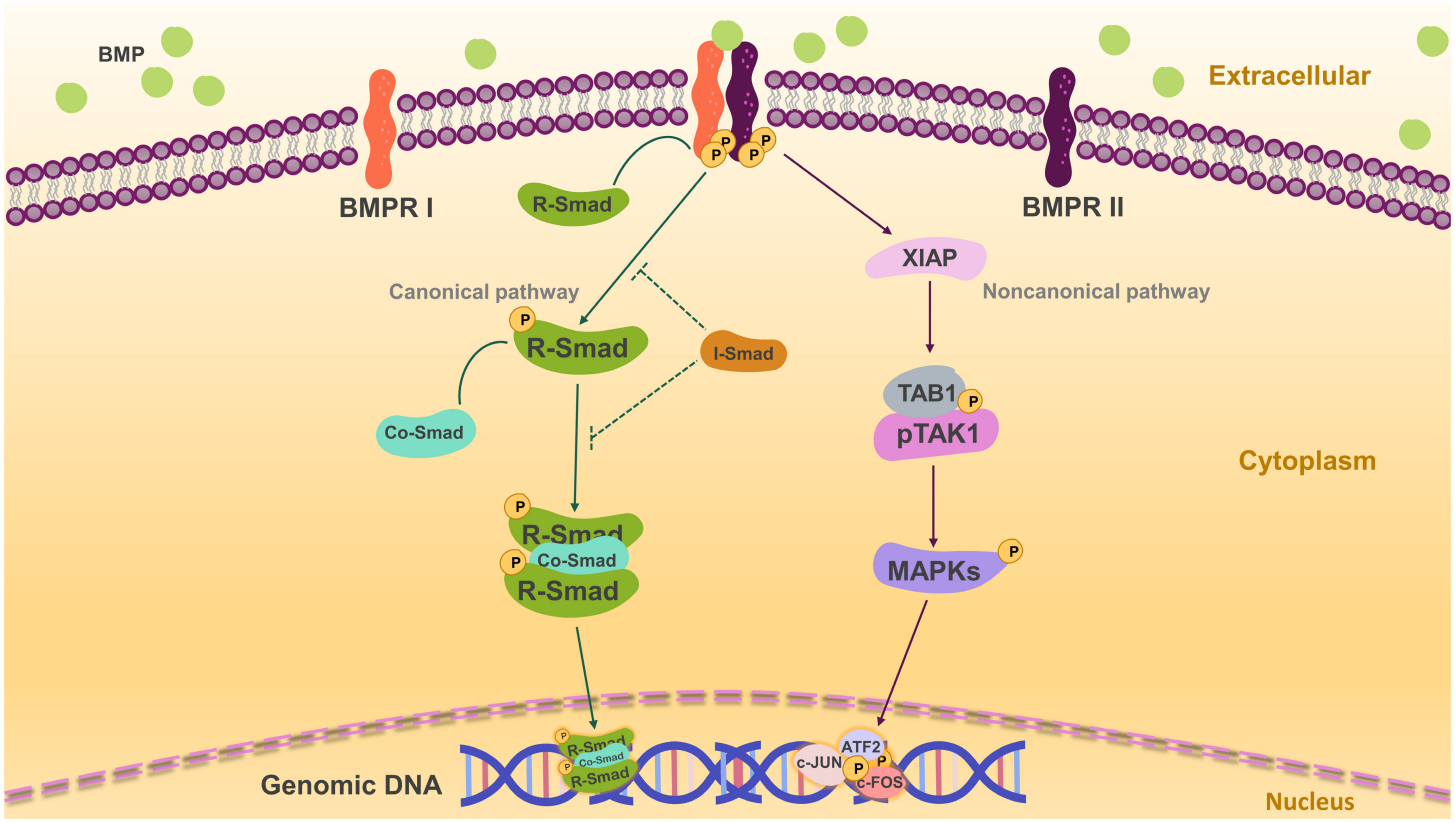
Bone morphogenetic protein (BMP) pathway signalling schematic. SMAD proteins perform the function of the canonical BMP signalling pathway. BMP ligands interact with combinations of Type 1 (BMPRI) and Type 2 receptors (BMPRII), activating effectors. BMP ligands interact with BMPRII to phosphorylate BMPRI. Under the phosphorylation of BMPRII, BMPRI phosphorylate downstream factors R‐SMADs (SMAD1/5/8), form a complex with co‐factor co‐SMAD (SMAD4), translocate into the nucleus and regulate the transcription of downstream target genes directly. Additionally, I‐SMADs (SMAD6/7) regulate the recruitment and activation of R‐SMADs. Noncanonical BMP signalling via TAK1 phosphorylation. BMPRI binds to TAK1‐TAB1 via XIAP, phosphorylates and activates downstream mitogen‐activated protein kinases (MAPKs). These active kinases subsequently translocate into the nucleus, phosphorylate and activate ATF2, c‐JUN and c‐FOS to complete gene control of genomic DNA.

The BMP signalling pathway transmits and performs cytological functions via Smad‐dependent classical pathways and numerous non‐classical pathways.^13^ In the canonical Smad‐dependent pathway, BMPs bind type II receptors (BMPR2), phosphorylate type I receptors (BMPR1 or ALK1‐7) and lastly phosphorylate the intracellular effector protein R‐Smads (Smad1/5/8).^14^ Activated R‐Smads form a protein complex with co‐Smad (Smad4) that enters the nucleus and regulates the transcription of downstream target genes in a direct manner. Moreover, the repressive I‐Smads (Smad6/7) inhibit the R‐Smad‐dependent TGF‐β signalling pathway by acting as repressors (show in Figure 1).

BMPs also have a noncanonical pathway, in which BMPR I binds to the TAK1‐TAB1 complex via X‐linked inhibitor of apoptosis (XIAP). The phosphorylated complex subsequently activates downstream mitogen‐activated protein kinases (MAPKs), such as p38, ERK1/2 and JNK. The phosphorylation of these kinases in the nucleus activates ATF2, c‐JUN and c‐FOS to regulate transcription of downstream target genes. In addition, BMP signalling regulates extracellular (e.g. noggin), intracellular (e.g. FKBP12, small RNAs, phosphatases and I‐Smads) and plasma membrane co‐receptors (e.g. endoglin) signalling pathways.^11^, ^15^, ^16^

## 3 BMP10 IN CARDIAC DEVELOPMENT AND CONGENITAL HEART DISEASE

As one of the earliest organs to form and develop during embryonic development, the heart is regulated by specific extracellular microenvironmental signals, including endoderm‐derived induction signals, cytokines and signalling pathways. BMP10, a secreted peptide, influences the development of the heart in an autocrine or paracrine manner. It is involved in the differentiation of cardiac progenitor cells, and its expression is selectively regulated by BAF250a (a constituent of the SWI/SNF complex),^17^ according to studies. Moreover, the combination of myocardin complex and CArG can activate the BMP10 gene, thereby regulating embryonic cardiomyocyte proliferation and chamber maturation.^18^

### 3.1 Trabeculation

Trabeculation serves a crucial role in the development of the heart. The dilation of ventricular trabeculae generates enough myocytes to form future septal, papillary and conduction system cells. It was discovered that BMP10 plays an important function in the development and maturation of cardiomyocytes as well as the formation of trabeculae in the embryonic heart.^19^ Participating in the formation of ventricular trabeculae and regulating the development and maturation of the ventricular wall, BMP10 induces the activity of Tbx20 (a T‐box family transcription factor) via a conserved Smad‐binding site.^7^ BMP10 affects another regulator involved in trabecular formation, Tie2, which supports endocardial cells and ensures normal trabecular formation. However, Tie2 also prevents excessive trabecular formation by inhibiting trabecular cardiomyocytes, and even its specific attenuation can lead to simplistic hyperplasia of the trabecular meshwork (less number but thicker), ultimately leading to fetal death in the second trimester.^20^

Despite the fact that regulatory factors such as BMP10 promote myocardial growth in the microenvironment, there are also negative cell cycle regulators that reduce myocardial proliferative activity, such as p57kip2 in trabecular myocardium, that regulate the equilibrium between myocardial proliferation and end‐stage differentiation. When this equilibrium is disrupted, cardiac hyperplasia, excessive trabecular formation and insufficient compaction result. FKBP12 (FK506‐binding protein 12, a cytoplasmic protein) negatively regulates BMP10 via interaction with BMP10 I type receptors, leading to elevated BMP10 expression in FKBP 12‐deficient myocardium or defective BMP10 leading to cardiac insufficiency, according to a separate study.^21^ Conversely, direct repression of BMP10 expression by the Irx gene is required during ventricular septal development and even influences postnatal cardiac functions, such as cardiac repolarization (Irx5) and rapid ventricular conduction (Irx3). Irx3 and Irx5 regulate their expression by binding directly to the BMP10 promoter in the endocardium, thereby maintaining normal ventricular septum development.^22^ Additionally, BMP10 is necessary for the formation of cardiac morphology. Pitx2 modulates the morphogenesis of the left–right asymmetry of the heart during fetal left atriogenesis by activating and repressing BMP10.^23^ Crossveinless‐2 [also known as BMP endothelial precursor‐derived regulatory protein (BMPER)] is a BMP‐binding protein mentioned in the study by Jumabay et al.^24^ CV2 regulates BMP10‐induced cell proliferation by directly binding to BMP10 and inhibiting Smad signalling initiated by BMP10.

### 3.2 Endocardial cushion development

By the fifth week of embryonic development, the heart shape has been essentially established, but the separation of internal tissues continues. Each endocardial cushion creates two bulges as a result of the proliferation of subendocardial tissue in the dorsal and ventral walls of the atrioventricular canal between the atria and ventricles. These two endocardial cushions develop in opposition to one another and fuse, thereby dividing the AV (atrioventricular) canal into the left and right atrioventricular foramen.^25^, ^26^ BMP10 is implicated in endocardial development by modulating the expression levels of cardiac‐derived transcription factors such as Nkx2.5 and Mef2c, which are downstream of the pathway. Further, while BMP10 in the endocardium stimulates the expression of Nkx2.5, Nkx2.5 can stimulate the expression of BMP10.^27^ Analysis of the Nkx2.5 promoter revealed that the expression of Nkx2.5 was regulated by numerous conserved Smad binding sites. This indicates that the BMP signalling pathway is implicated in the entire cardiac development process, including the initial induction of cardiogenesis. Through the Smad‐Nkx2.5 pathway, it is highly plausible that BMP10 plays an important part in maintaining the homeostasis of cardiac function. Researchers discovered a rapid decrease in Nkx2.5 and Mef2c levels in BMP10‐lacking hearts. In contrast, in the hearts of mice lacking Nkx2.5, BMP10 expression was upregulated ectopically. All of these findings suggest that BMP10 may play a significant role in ventricular maturation via the Smad‐Nkx2.5 pathway.

In addition, epithelial–mesenchymal transition (EMT), a crucial stage in the development of the endocardial cushion, occurs in a portion of endocardial cells in the atrioventricular canal, which eventually forms a heart valve. Throughout this process, BMP10 promotes EMT. Compared to hearts with normal cushion structure, the endocardial cushions of BMP10‐deficient hearts lacked mesenchymal cells, resulting in aberrant endocardial cushion development.^21^

### 3.3 Physiological hypertrophy of the heart muscle

As part of the normal developmental process, the vast majority of cardiomyocytes experience a rapid decrease in cell cycle activity and cease proliferating at the completion of terminal differentiation and maturation. This withdrawal from the cell cycle is believed to play a crucial role in the transition of cardiac cells from proliferative to hypertrophic growth. In general, this phenomenon of physiological hypertrophy of the myocardium utilizes haemodynamics independently or to a lesser extent to exert burden. BMP10 operates as a potential modifier of cell cycle activity changes, among other functions. If BMP10 is consistently expressed at high levels in the embryo, the cell cycle activity of cardiomyocytes is also maintained at high levels, leading to aberrant ventricular wall development characterized by excessive myocardial trabeculation and compaction insufficiency. In order for the ventricular myocardium to develop in a normal physiological state, the expression of BMP10 must be downregulated in late embryonic development. It is now evident that BMP10‐mediated signalling pathways are not only essential for embryonic heart development, but also regulate postnatal myocardial growth and maturation.^28^ Compared to the intracellular signalling pathways involved in pathological myocardial hypertrophy, physiological myocardial hypertrophy is associated with two main intracellular signalling pathways. The BMP‐Smad signalling pathway may mediate the growth of postnatal myocardial hypertrophy and function as a parallel or downstream pathway of the PI3K‐Akt pathway, which together influence the growth and regulation of postnatal physiological hypertrophy.

It is believed that Brg1, a chromatin protein that maintains the embryonic state of cardiomyocytes, promotes cardiomyocyte proliferation by maintaining BMP10 levels in the embryo and inhibiting p57^kip2^ expression. According to a number of studies, adult cardiomyocytes predominantly express α‐MHC (α‐myosin heavy chain, also known as Myh6), whereas embryonic cardiomyocytes express β‐MHC (β‐myosin heavy chain, also known as Myh7). By inhibiting α‐MHC and activating β‐MHC through interaction with histone deacetylase (HDAC) and poly ADP ribose polymerase (PARP) during embryogenesis, Brg1 can maintain regular differentiation of the foetal heart. Brg1 also referred to as Smarca4, is typically inactive in adult cardiomyocytes. Once the heart is stressed, Brg1 is reactivated and forms a complex with HDAC and PARP, inducing a transition from α‐MHC in cardiomyocytes to β‐MHC in the foetus and ultimately leading to the pathological myocardial hypertrophy frequently observed in adult CVD.^29^ Thus, Brg1 regulates cardiac growth, differentiation and gene expression. Preventing the re‐expression of Brg1 may rectify the MHC transition by affecting downstream BMP10, thereby ameliorating cardiac hypertrophy symptoms. In pathological conditions of hypertension, in addition to the Brg1‐BMP10‐MHC pathway of cardiac hypertrophy, BMP10 can interact with the key functional protein titin‐cap (Tcap) on the cell surface of cardiomyocytes and in the Z‐disc.^30^ Considering that hypertension is a major risk factor for the development and progression of CVD, it can induce myocardial hypertrophy by increasing wall pressure and releasing angiotensin II, among other mechanisms. It is not difficult to comprehend that the expression of pro‐hypertrophic BMP10 was elevated in the ventricles of hypertensive model rats and that this protein regulates myocardial hypertrophy by binding to Z‐disc Tcap. All of these findings offer novel insights into the investigation of CVDs characterized by myocardial hypertrophy.

### 3.4 Congenital heart disease (CHD)

The development of the heart is regulated by a complex network of signals, and variations in intrinsic genes or the external environment may influence particular signalling pathways. While we have described the normal physiological expression of BMP10 in the embryonic heart, some research indicates that it may also play a role in certain pathological conditions. The Notch signalling pathway has a strong connection with the formation of cardiac atrioventricular tubes, valves, and muscle trabeculae and mutations in its key molecules can result in irreversible cardiac defects. As one of the key molecules of the Notch signalling pathway, the upregulation of BMP10 expression promotes cardiomyocyte proliferation, but also causes trabecular hyperplasia in the ventricle and ventricular wall compaction insufficiency. In cardiomyocytes with mutations in the Notch‐dependent gene V407I‐BMP10, the binding of mutant BMP10 to BMPR1a and BMPR2 was diminished. This may be because the V407I variant of BMP10 interferes with BMP protein receptor binding and the function of cell proliferation and differentiation, ultimately resulting in LV wall compaction.^31^

Additionally, BMP10 is involved in the pathological process of patent ductus arteriosus (PDA) in neonates, and the synergy of BMP9 is indispensable.^32^ This may be owing to a chromosome q31.3 deletion caused by mutations in MYCN downstream of BMP10 signalling. MYCN expression promotes normal myocardial wall morphology under normal conditions, and its expression is double‐regulated by the BMP10 and NRG1 signalling pathways.^33^ However, disruption of either the BMP10 or NRG1 pathways results in developmental disorders marked by cardiac defects, including the aforementioned fetal patent ductus arteriosus.

## 4 BMP10 IN CARDIOVASCULAR DISEASE

### 4.1 Myocardial infarction and myocardial injury

Globally, CVD is a leading cause of morbidity and mortality. Myocardial cell injury is generally regarded as the primary symptom of CVDs, such as myocardial infarction (MI). Myocardial infarction is irreversible myocardial necrosis brought on by protracted ischemia. In recent years, numerous studies have indicated that the induction of cardiomyocyte proliferation is a potential treatment for cardiac diseases.^34^, ^35^, ^36^ As a key player in the development of the heart, BMP10 also regulates the proliferation and apoptosis of cardiomyocytes.

Not surprisingly, in the exploration of CVDs, BMP10 has caught the attention of scientists. After observing rat models of myocardial infarction, they discovered that exogenous BMP10 stimulated cell cycle re‐entry and mitosis in post‐infarct cardiomyocytes, resulting in a reduction in infarct size and enhanced cardiac repair.^37^ Thus, BMP10 can stimulate cardiomyocyte proliferation and restore damaged cardiac function. This discovery applies not only to myocardial infarction, but also to the treatment of damaged cardiomyocytes caused by doxorubicin (DOX)‐induced cardiac injury. Although DOX exposure decreased the initial level of BMP10 in the heart, exogenous supplementation may ameliorate DOX‐induced systolic dysfunction. DOX cardiotoxicity decreased phosphorylation of signal transducer and activator of transcription 3 (STAT3), and exogenous BMP10 reversed the defective STAT3 via a noncanonical pathway.^38^ Hence, it is evident that BMP10 has the capacity to elicit cardiomyocyte value‐added and thereby counteract cardiac damage, both for cell death due to myocardial ischemia and cardiac injury induced by drugs, and that this capacity might be achieved via the STAT3 pathway.

### 4.2 Atrial fibrillation

Arrhythmias, another important group of CVDsymptoms, can occur alone or in conjunction with other CVDs. High‐risk factors such as hypertension, diabetes and obesity can result in electrical and structural changes to the atria. Atrial fibrillation (AF), one of the most prevalent of these arrhythmias, can contribute to the deterioration of cardiac function in primary CVDs such as heart failure and myocardial ischemia.

As previously described, BMP10 is involved in the growth and development of cardiomyocytes and is essential for cardiac function throughout all stages of human development. In recent years, researchers have discovered that the level of BMP10 expression is consistently elevated in patients with persistent AF.^39^ When AF is ablated and sinus rhythm is restored, the level of BMP10 decreases and declines significantly.^40^ Evidently, BMP10 is substantially associated with persistent atrial fibrillation and may serve as a biomarker for early detection of atrial fibrillation in clinical practice. How then does BMP10 contribute to the pathogenesis of AF Steimle et al. hypothesized that BMP10 transmits pathogenic signals from cardiomyocytes containing mutant Pitx2, which encodes a critical transcription factor in development, to endothelial and endocardial cells in the pulmonary vein and left atrium. Normal expression is regulated by a non‐coding AF‐associated region (AFAR) at the 4q25 locus, which is frequently associated with the development of AF. When the AFAR gene is removed in mice, the expression of Pitx2 is substantially reduced, whereas the expression of BMP10 and inflammatory factors is increased, resulting in an increased susceptibility to AF in humans.^41^ Furthermore, AF is frequently caused by ectopic electrical impulses generated in the pulmonary capillaries, suggesting that BMP10 has the potential to be more than an early warning marker for AF and may provide new insights into the pathogenesis of AF.

## 5 EFFECT OF BMP10 ON VASCULAR ENDOTHELIUM

### 5.1 Regulation of vascular endothelium

Simultaneous expression of functionally equivalent ligands can result in functional redundancy in the BMP signalling pathway.^42^ BMP9 and BMP10 are two high‐affinity ligands of activin receptor‐like kinase 1 (ALK1) signal transduction,^43^ and their physiological functions, specifically modulating angiogenesis and remodelling, are equivalent.^8^ The two are connected by disulphide bonds to form homodimers and heterodimers, respectively. By way of ALK1, circulating heterodimers of BMP9/BMP10 are the predominant active form on endothelial cells.^44^ In addition to ALK1, the activation of BMP9/BMP10 dimer requires BMPR2 and endoglin (antigen associated with cell proliferation, which is required for angiogenesis) to form a specific receptor complex that regulates the differentiation of vascular endothelium in endothelial cells and morphogenesis, thereby preserving the equilibrium of vascular growth and maturation of lymphatic vessels.^45^ ALK1, a key Type 1 receptor that regulates the SMAD phosphorylation pathway and is encoded by ACVRL1, is almost exclusively expressed on vascular endothelial cells in the endothelium, mediating normal tissue and tumour angiogenesis. When signalling at the ALK1 locus is inhibited, vascular endothelial growth factor, fibroblast growth factor and BMP10‐mediated angiogenesis are inhibited, thereby reducing vascular growth and lymphatic vessel maturation.^43^ Downstream of this BMP9/BMP10‐ALK1 signalling pathway, the endothelial‐specific protein Tmem100 participates in the formation of embryonic valves and septa.^46^ The deletion of the Tmem100 gene reduces the activity of Notch and Akt‐mediated signalling pathways, resulting in impaired arterial endothelial differentiation and vascular morphology defects in embryos.^47^

In circulation, biologically active BMP9 and BMP10 can heterodimerize through disulphide bonds and thus exist in both homodimeric and heterodimeric forms as ligands. These proteins are processed by convertases into a proprotein domain and a mature growth factor domain, and the cleaved prodomain forms an active complex known as BMP‐pd by non‐covalently binding to the mature domain.^44^ Previous study has demonstrated that BMP9 is only expressed in the liver and is produced by hepatic stellate cells. Additionally, these hepatic stellate cells can express BMP10.^45^ Unfortunately, it is unknown whether BMP10, which forms an active dimer with BMP9 in the bloodstream, originates from the liver, the heart or both. Together with BMP 9, BMP10 has been implicated in regulating the phenotype of vascular smooth muscle cells (VSMCs).^48^ Studies on adult mice have demonstrated that BMP10 can act directly on VSMCs in developing atria, inducing and maintaining their contractile state phenotype. In the pulmonary circulation, the BMP9/10 complex can induce VSMCs in a specific manner via BMP type 1 receptors (i.e. ALK1, ALK2, ALK3 and ALK6) at various vascular bed sites.^49^ And inactivation of any of them can substantially induce a phenotypic shift from the contractile to the synthetic state of VSMC, resulting in a decrease in VSMC layer and a decrease in systemic blood pressure.

It is known that BMP9 functions as an angiostatic factor in the vascular endothelium, maintaining the vascular endothelium in a dormant state and preventing neovascularization, thereby maintaining the vasculature in a quiescent state. Similarly, it has been reported that BMP10 mediates vascular quiescence in zebrafish.^5^ Beyond that, some researchers have hypothesized that blood flow is crucial for the modulation of BMP10 in arterial endothelial cells. During embryonic vascular development, circulating BMP10 can stabilize the diameter of embryonic arteries by limiting the number of endothelial cells via the ALK1 protein in endothelial cells. Blood flow promotes ALK1 activity and induces ALK1 expression and BMP10 distribution in this process. In contrast, blood flow and BMP10 increased ALK1 activity synergistically.^50^ Interestingly, BMP10 has biological effects on micro‐vessels as well. In the absence of BMP9, BMP10 can replace its function and participate in the regulation of retinal angiogenesis, according to mouse model research.^51^

### 5.2 Vascular endothelial dysfunction

Previously, we described how BMP10 is involved in the regulation of the vascular endothelium and its functions. However, if either ligand or receptor is absent from this pathway, it can result in a variety of vascular dysfunctions. Mutations in any of the receptor complexes, that is, ALK1, BMPR2 or endoglin, can result in pathological alterations in normal blood vessels, such as idiopathic and hereditary pulmonary hypertension (PAH).^52^ As a ligand of ALK1 (a type I BMP receptor) with high affinity, mutations in BMP10 can cause PAH.^53^, ^54^, ^55^ Therefore, the BMP9/BMP10‐ALK1 axis is particularly crucial for the integrity and functionality of pulmonary vascular endothelial cells.

In recent years, a rare gene mutation, GDF2 mutation, has been identified in PAH patients, resulting in decreased levels of circulating BMP10 and BMP9, which impairs cellular processing and secretion functions.^56^, ^57^ Therefore, it is conceivable that GDF2 regulates the expression of BMP10 and BMP9 in the circulation as a master gene. Also, as the decrease of BMP10 in plasma of female patients is particularly pronounced, it is possible that gender also influences this process.

If the ligand BMP10 in the BMP9‐BMP10‐ALK1 pathway is mutated, it will result in abnormal blood vessels in the epidermis and liver, as well as hereditary haemorrhagic telangiectasia (HHT).^45^, ^58^ Ruiz et al. demonstrated that the absence of BMP10 increases the probability of developing HHT. They injected the anti‐BMP9/BMP10 antibody intraperitoneally into the lactating mother mice because the antibody can enter the blood circulation of the baby mice via breastfeeding, resulting in vascular lesions consistent with those of the mother on the baby mice's retina, including vascular hyperplasia and arteriovenous functional deficits, as well as numerous arteriovenous malformations.^59^ According to the pathogenesis of HHT, endothelial cells exhibit excessive activation of the PI3K/Akt/mTOR and VEGFR2 pathways. In another study, this BMP9/BMP10 immune blockade (BMP9/10ib) HHT model was established in young mice using the inhibitors sirolimus and nintedanib to block hyperactivation of mTOR and VEGFR2.^60^ The findings demonstrated that this combination reversed retinal AVMs, prevented anaemia and retinal haemorrhage symptoms, and prevented vascular lesions in the oral mucosa, lung and liver of young mice.

In a study of arterial duct (DA) closure, it was discovered that EMT and endothelial‐to‐mesenchymal transition (endMT) occur, during which BMP9 and BMP10 play a crucial and irreplaceable role by strongly inducing mRNA upregulation of transcription factors SNAI1, SNAI2, ZEB2, TWIST1 and FOXC2. Besides, BMP9 and BMP10 induce the transient expression of transcription factors HEY1, HEY2 and HES1 of the Notch signalling pathway, which are implicated in the EMT process. Moreover, fibronectin, a crucial component of DA closure, is one of the matrix proteins that surround endothelial cells. In DA and human pulmonary artery endothelial cells (HPAECs), BMP9 and BMP10 strongly induce fibronectin expression, but not Type I collagen expression.^32^

## 6 BMP10 IN IMMUNOLOGICAL INFLAMMATION AND OXIDATIVE STRESS

One of the key mechanisms leading to microvascular endothelial dysfunction is the inflammatory immune response. If inflammatory factors are disrupted, apoptosis and cell injury will be induced, which will result in impaired microvascular endothelial function and promote the onset of diabetes. As the most prevalent innate immune cells in adipose tissue, macrophages play an important role in the metabolic inflammation that leads to T2DM. Increasing evidence suggests that BMP9 and BMP10, besides participating in the normal physiological processes of the cardiovascular system, may maintain endothelial homeostasis by limiting the monocyte/macrophage system while permitting inflammatory responses under certain conditions. In response to inflammatory stimuli, BMP9 and BMP10 upregulate the expression of the inflammatory factors E‐selectin, VCAM‐1 and ICAM‐1 via the ALK2 type I receptor, the BMPR‐II/ACTR‐IIA type II receptor and the downstream Smad1/5 signalling pathways. In the presence of TNF‐α, the beneficial role of BMP10 as a vasostatic factor can be disrupted, leading to an increase in the recruitment of monocytes to the vascular endothelium and a promotion of the inflammatory response. Intriguingly, BMP10 or BMP9 alone have no effect on monocyte recruitment, and only at higher concentrations can BMP9 and BMP10 synergize with TNF‐α to increase recruitment of monocytes in the vascular endothelium and induce BMP2 expression, thus indirectly contributing to the initiation of an inflammatory response.^61^

On the other hand, BMP10 can inhibit the basal expression and release of another inflammatory factor, CCL2, in endothelial cells at specific sites, such as the pulmonary arteries and aorta. CCL2 is one of the main chemokines that regulates the migration and infiltration of monocytes and macrophages. It has chemotactic activity on inflammatory cells and contributes to the development of CVDs such as atherosclerotic plaque and PAH.^62^ BMP10 can cooperate with BMP9 to bind to ALK1 and exert inhibitory effects on CCL2 via the Smad1/5 and Smad4 pathways, according to research.^63^ Beyond that, ATF3, a downstream target of BMP10, exerts a stress response via the Smad pathway, thereby contributing to the immune response.^64^ As a member of the ATF/CREB family, ATF3 has a stress‐inducing function and can respond in vivo and in vitro to numerous stimuli, such as oxidative stress.

## 7 BMP10 IN DIABETIC CARDIOMYOPATHY

Diabetic cardiomyopathy (DCM) is primary cardiomyopathy, a disease that excludes coronary artery disease, hypertension and currently known cardiac diseases. It is characterized by abnormal cardiac diastolic function, ultimately resulting in whole‐heart diastolic and systolic dysfunction and the development of heart failure. In a study of molecular transcript levels in diabetic cardiomyopathy, researchers analysed changes in the levels of various cardiac transcription molecules using an Akita mouse model of diabetes with cardiac dysfunction and found that BMP10 expression was the most upregulated of all 137 groups.^65^ This finding suggests that BMP10 may be a candidate gene that will significantly advance our understanding of the molecular mechanisms underlying DCM.

Endothelial dysfunction, inflammatory immune response and oxidative stress all have important effects on the development of DCM, causing damage to cardiomyocytes, fibroblasts and endothelial cells and ultimately severe pathological myocardial remodelling.^66^, ^67^ Usually, cardiac myocytes, fibroblasts and endothelial cells are affected by this pathological change. These pathological alterations frequently manifest as myocardial hypertrophy and fibrosis. In a study of pregnant women with gestational diabetes, it was found that hyperglycaemia can cause an increase in cardiomyocyte size and a concomitant increase in protein synthesis, resulting in myocardial hypertrophy in the fetus during pregnancy. Significant inhibition of the cardiogenic inducer Nkx2.5 limits the expression of the downstream genes KCNE1 and Cx43. In conjunction with the production of excessive reactive oxygen species (ROS), these factors contribute to the eventual development of prenatal neonatal myocardial hypertrophy. Therefore, abnormal expression of BMP10‐ and Nkx2.5‐regulated target genes in a high‐glucose environment may be a significant contributor to the pathogenesis of myocardial hypertrophy.^68^

**FIGURE 2:**
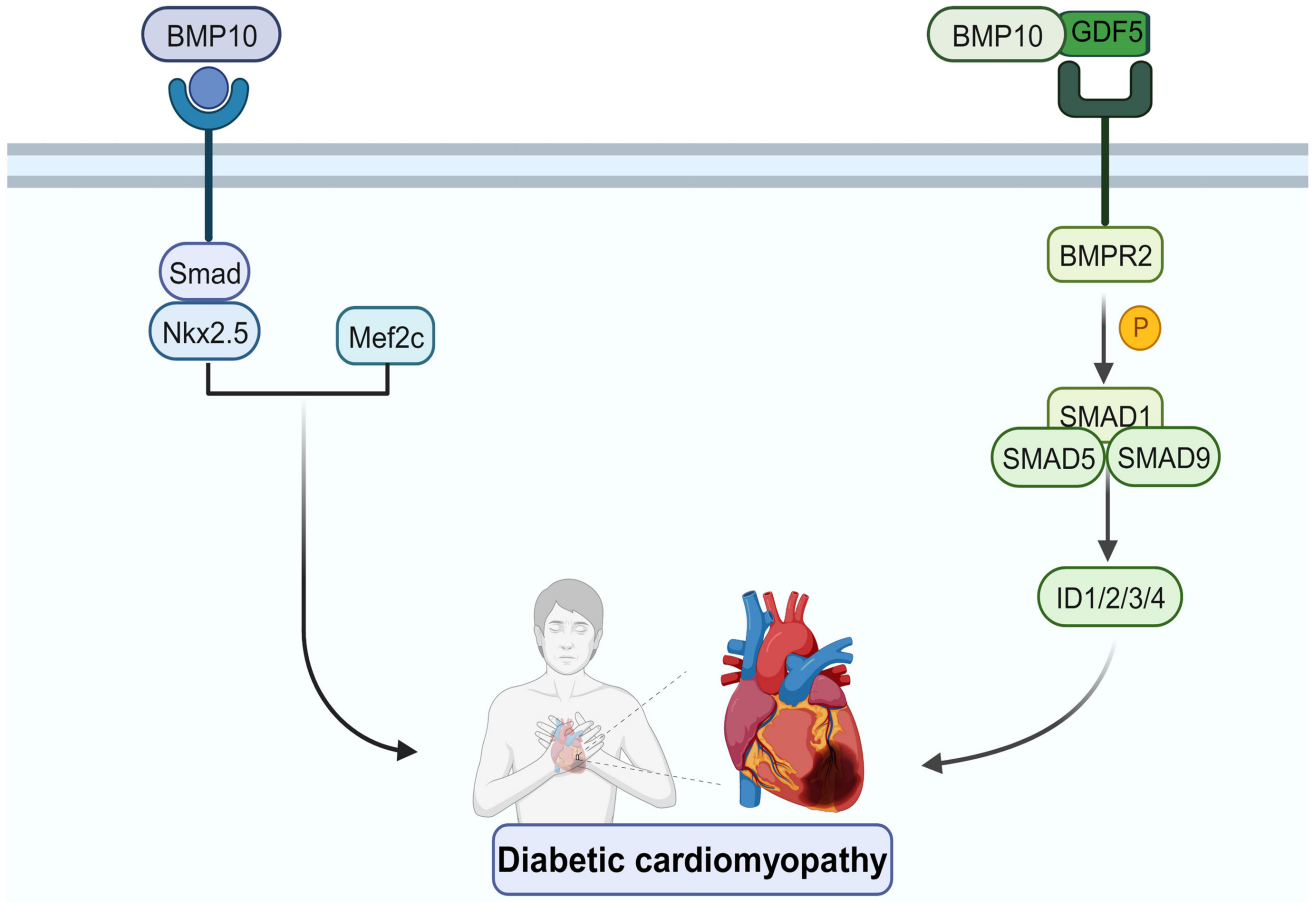
BMP10 role in pathogenesis of diabetic cardiomyopathy. BMP10 plays a role in diabetic cardiomyopathy. BMP10 is involved in endocardial development by regulating the expression levels of Nkx2.5 and Mef2c downstream of this pathway.^69^, ^70^ The growth differentiation factor GDF5 can form a complex with BMP10, with the regulatory downstream SMAD1/5/8, translocate into the nucleus and directly regulate the transcription of downstream target genes.

Myocardial hypertrophy is purportedly the defining characteristic of diabetic cardiomyopathy and is intricately linked to cardiac dysfunction.^69^ Specific cardiac peptide growth factor BMP10 is a critical contributor to cardiac hypertrophy.^21^ During embryonic development, BMP10‐deficient mice develop ventricular hypoplasia and feeble hearts, whereas BMP10‐overexpressing mice develop excessive ventricular trabeculae and myocardial hypertrophy.^27^, ^70^ Furthermore, BMP10 is involved in high glucose‐induced myocardial hypertrophy.^68^ Based on these results, it can be concluded that BMP10 significantly influences the progression and initiation of myocardial hypertrophy. BMP10 functions by phosphorylating downstream factors Smad1/5/8; Tbx20, Nkx2‐5 and Mef2c are among the activated genes that regulate hypertrophy.^18^ BMP10, ALK3, BMPRII and p‐Smad1/5/8 expression were all upregulated in hyperglycaemia‐stimulated cardiomyocytes and diabetic cardiomyopathy rodents, according to the study (Figure 2).

Myocardial fibrosis, characterized by myocardial remodelling, impaired cardiac function and increased ventricular rigidity due to fibroblast aggregation and excessive extracellular matrix deposition, is another pathological myocardial remodelling in DCM. BMP10 is known to play an essential part in the protection of adult myocardium and the development of the embryonic heart, but its role in myocardial fibrosis is unknown. Overexpression of BMP10 results in excessive ventricular hypertrophy and excessive trabecular proliferation during embryonic heart development, whereas deletion of BMP10 results in embryonic mouse mortality due to failure of heart development. Meanwhile, BMP9 knockdown appeared to have no effect on the normal development of the rodent heart.^71^ This suggests that BMP10 is potentially beneficial in alleviating cardiac fibrosis and that deleting BMP10 may increase the risk of myocardial fibrosis.

It has been demonstrated that the growth differentiation factor GDF5 can form a complex with BMP10, and that the N‐terminus of fibrillin‐1 (a component of the microfibril network that interacts with TGF‐β) is the high‐affinity interaction site between the two.^72^ Fibrillin is an extracellular microfibrillar protein produced by fibroblasts that regulates the physiological functions of connective tissue. The prodomain of BMP10 inhibits its own biological activity, and the BMP10‐GDF5 complex is activated by breaking the prodomain and binding to the N‐terminus of fibrillin, thereby influencing the downstream connective tissue pathways.^73^ Additionally, a study of cirrhotic patients revealed that BMP10 was associated with liver fibrosis in cirrhotic decompensation patients. Reduced plasma levels of BMP9 and BMP10, as well as reduced mRNA expression, led to a decrease in serum BMP activity in the vascular endothelium. In contrast, patients with compensated cirrhosis or lesser levels of liver fibrosis had normal plasma levels of BMP9 and BMP10.^74^ All of these evidences suggest that BMP10 is implicated in the pathogenesis and progression of diabetic cardiomyopathy, particularly the two myocardial remodels, myocardial hypertrophy and myocardial fibrosis.

## 8 DISCUSSION

As one of the complications of diabetes, diabetic cardiomyopathy has a complex pathogenesis. It manifests both coronary microcirculatory lesions characterized by endothelial dysfunction and triggers abnormal cardiac function characterized by cardiomyopathy, including abnormal manifestations such as myocardial hypertrophy, myocardial fibrosis, extensive myocardial necrosis and abnormal electrical activity of the heart.

From our perspective, BMP10 is implicated in multiple aspects of the development and progression of diabetic cardiomyopathy. The synergistic role of BMP10 and BMP9 in inducing endothelial maturation and differentiation in the vascular endothelium has been discussed more frequently in recent years. Sadly, the role of BMP10 has not been described in published articles, let alone in studies examining the interaction between diabetes and cardiovascular mechanisms. In this paper, we look into the effects of BMP10 on cardiac development, CVD and endothelial function. This, we believe, will enable us to comprehend more systematically how BMP10 functions in the complex mechanism involving multiple factors in diabetic cardiomyopathy. Unexpectedly, it also serves a subtle part in the induction of immune inflammation and oxidative stress. This strengthens our conviction that BMP10 may offer novel insights into the study of diabetic cardiomyopathy.

However, we discovered that research on BMP10 is limited compared to other members of the BMP family, and that there are insufficient reports clarifying the functions and specific mechanism of action of BMP10. Therefore, we hope that this review will motivate more researchers to focus on BMP10 research, so as to further encourage the investigation of the mechanism of diabetic cardiomyopathy and the development of effective solutions for future clinical treatment areas.

## AUTHOR CONTRIBUTIONS

**Helin Sun:** Formal analysis (equal); methodology (equal); validation (equal); visualization (equal); writing – original draft (equal); writing – review and editing (supporting). **Xueyin Wang:** Formal analysis (equal); methodology (equal); validation (equal); visualization (equal); writing – original draft (equal); writing – review and editing (lead). **Haomiao Yu:** Data curation (equal); validation (supporting). **Bingyu Du:** Data curation (equal); validation (supporting). **Qi Fan:** Data curation (equal); validation (supporting). **Baoxue Jia:** Methodology (supporting); project administration (supporting); supervision (supporting); writing – review and editing (supporting). **Zhongwen Zhang:** Conceptualization (lead); funding acquisition (lead); project administration (lead); resources (lead); supervision (lead).

## CONFLICT OF INTEREST STATEMENT

The authors confirm that there are no conflicts of interest.

## Data Availability

The data that support the findings of this study are available from the corresponding author upon reasonable request.
